# Re-examining the crystal structure behaviour of nitrogen and methane

**DOI:** 10.1107/S2052252520007460

**Published:** 2020-07-29

**Authors:** Helen E. Maynard-Casely, James R. Hester, Helen E. A. Brand

**Affiliations:** a Australian Nuclear Science and Technology Organisation, Locked Bag 2001, Kirrawee DC 2232 Australia; b Australian Synchrotron, ANSTO, 800 Blackburn Road, Clayton 3168, Australia

**Keywords:** molecular crystals, Pluto, thermal expansion, phase transitions, solid properties, methane, simple crystal structures

## Abstract

In the light of NASA’s *New Horizons* mission, the solid-phase behaviour of methane and nitrogen has been re-examined and the thermal expansion coefficients of both materials have been determined over their whole solid temperature range for the first time. Neutron diffraction results indicate that the symmetric *Pa* 3 space group is the best description for the α-nitrogen structure, rather than the long-accepted *P*2_1_3. Furthermore, it is also observed that β-nitrogen and methane phase I show changes in texture on warming, indicating grain growth.

## Abstract 2

In an effort to better understand Plutonian glaciology, the thermal expansion of solid methane and nitrogen have been determined using neutron powder diffraction. Additional results include a resolution of the long-standing dispute over the α-nitrogen crystal structure, and observations of β-nitrogen texture change with temperature.

## 1. Introduction   

This study is motivated by the striking images returned from the *New Horizons* fly-by of Pluto (Stern, 2009 ▸), including images of towering mountains surrounded by seemingly flowing terrain (Moore *et al.*, 2016 ▸). The explanation for this terrain has its base in crystallography, where at 44 K the strength of the hydrogen bond endows water–ice with the resilience to build such mountains, while the rotational disorder in methane (Press, 1972 ▸) and nitrogen (Press *et al.*, 1982 ▸) allows these materials to flow at cryogenic temperatures. These interpretations have been strengthened by spectral observations that correlate these materials to the respective terrains (Grundy *et al.*, 2016 ▸). There is much still to be understood about the interactions and possible new structures that form under these cryogenic temperatures, and crystallographic investigations play a key role.

The low-temperature structures of both methane and nitrogen have previously garnered significant interest, but these investigations tailed off thirty years ago with emphasis being more recently on their high-pressure structures (Neumann *et al.*, 2003 ▸; Maynard-Casely *et al.*, 2010 ▸, 2014 ▸; Hanfland *et al.*, 1998 ▸; Stinton *et al.*, 2009 ▸). In light of the fact that methane and nitrogen have now been confirmed to be important materials for shaping the surface of other planetary bodies, we undertook an investigation to examine their solid-phase behaviour and to determine their thermal expansion. Previous determinations of the thermal linear expansion of methane and nitrogen used a dilatometer, restricted to the range 4.2–40 K for nitrogen (Heberlein *et al.*, 1970 ▸) and 4–26 K for methane (Heberlein & Adams, 1970 ▸; Manzhelii *et al.*, 1969 ▸). In the present study, we determined the volume change and derived the linear expansion from neutron diffraction data, encompassing the whole range of Pluto temperatures (24 to 54 K) while also allowing insights into their crystal structures.

## 2. Experimental procedure   

The experiments on methane and nitrogen were conducted on the high-intensity diffraction instrument WOMBAT (Studer *et al.*, 2006 ▸) at the Australian Centre for Neutron Scattering. The instrument was set up with a single-crystal Ge [335] monochromator, which results in better peak resolution than the more regularly used focusing Ge [113] monochromator. The [224] monochromator reflection was used, with a wavelength of 1.641 (1) Å determined from refinement against data collected (https://doi.org/10.5281/zenodo.3637634) from NIST LaB_6_ 660b standard reference material.

Sample preparation began with a small amount (∼0.1 g) of NIST silicon 640c spread into silica wool and placed inside a 6 mm vanadium can. The can was sealed onto a gas delivery stick (Lee *et al.*, 2016 ▸) and placed within an orange cryofurnace (Brochier, 1977 ▸) mounted on the WOMBAT instrument. Methane (Coregas, 99.995%) and nitrogen (BOC, ultra-high purity) were separately condensed into the sample area and the diffraction data were collected (https://doi.org/10.5281/zenodo.3634152 and https://doi.org/10.5281/zenodo.3635106) in temperature steps starting at the base temperature (8 K for methane and 6 K for nitrogen) and increasing in 2 K steps until they each melted.

Methane and nitrogen lattice parameters were extracted with Pawley refinements (Pawley, 1981 ▸) implemented within the program *TOPAS 5.0* (Coelho, 2007 ▸). Multiple methods were used to improve the accuracy of the determined lattice parameters. Detector zero error, wavelength and instrumental peak widths were refined from a room-temperature pattern of NIST LaB_6_ 660b standard reference material (https://doi.org/10.5281/zenodo.3637634). Sample displacement within the cryostat was refined from the first pattern in each sequence, with variation in this value arising from the gas delivery stick position and absorption of each sample. For subsequent refinements at each temperature the lattice parameters and crystallite size [derived from the Lorentzian contribution to peak shape (Scardi *et al.*, 2018 ▸)] were allowed to vary during refinement.

In order to refine the α-nitrogen structure a second data set was taken at a wavelength of 2.413 (1) Å (https://doi.org/10.5281/zenodo.3635103). Rietveld refinements (Rietveld, 1969 ▸) using these data were performed with the *TOPAS* suite. This additional data collection from nitrogen was used to obtain information on the preferred orientation observed for β-nitrogen.

## 3. Results and discussion   

### 3.1. Methane   

#### 3.1.1. Thermal expansion of methane between 8 and 82 K   

**Figure 1:**
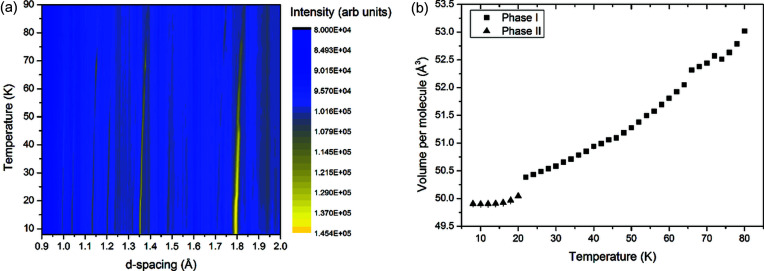
(*a*) Thermodiffractogram of neutron powder data collected on methane (CH_4_) between 8 and 82 K. (*b*) Extracted volume per molecule from a Pawley fitting of the methane diffraction. Error bars in the measurement are plotted but are in practice smaller than the data points. The scatter in the points at higher temperatures can be attributed to grain growth in the sample, which is supported by the change in intensities in (*a*) at these temperatures. The fitted lattice parameters used to generate (*b*) are tabulated in the supporting information.

Diffraction from methane (CH_4_) carries with it the consequences of undertaking neutron diffraction with hydrogenous material, that is, the lower signal-to-noise ratio due to the high background from the strong incoherent scattering of hydrogen. However, rather than studying deuterated methane, which could exhibit isotopic effects and therefore be less relevant to the Plutonian surface, we have taken advantage of the high flux of neutrons offered by the WOMBAT instrument to study hydrogenous methane. This meant our data had limitations, and additional Fourier filtering processing was applied to remove features arising from the detector response. The resultant thermodiffractogram and fitted expansion in volume per molecule is presented in Fig. 1 ▸.

As can be seen in Fig. 1 ▸ and has been observed before, methane has two distinct temperature-dependent solid phases at ambient pressure – methane I 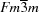 with 4 molecules per unit cell and methane II 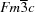 with 32 molecules per unit cell (Press, 1972 ▸). Phase I is the higher temperature form; the prevailing crystal structure model of this phase involves the methane molecules exhibiting full rotational disorder and the resulting spheres arranged in a cubic close-packed structure. The methane phase I structure persists to 20.4 K; below this temperature the methane molecules partially order to form phase II. The model that best describes this structure has 8 of the methane molecules retaining full rotational disorder, and the remaining 24 ordered molecules arranged about the disordered ones. The high background from the incoherent scattering meant that we were unable to distinguish between phases I and II from their diffraction patterns. However, as we extracted the volume from our Pawley fitting of the data, a clear step change in the volume per molecule between methane II and I can be seen in Fig. 1 ▸(*b*).

**Figure 2:**
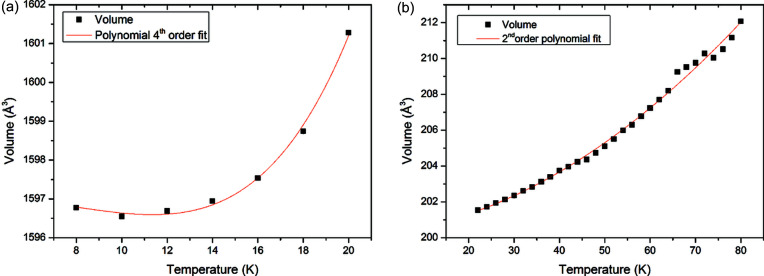
The polynomial fits to volume data from (*a*) methane phase II and (*b*) methane phase I. The fitted models are described in the text, and the fitted lattice parameters used to generate these figures are tabulated in the supporting information.

Volume data for methane Fig. 1 ▸(*b*) are tabulated in the supporting information. When fitting the volume data, we examined each phase separately and have presented the most appropriate polynomial expression to describe our data. Volume data for methane II between 8 and 20 K extracted from the data presented in Fig. 1 ▸(*a*) were fit to a fourth-order polynomial as follows:
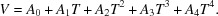
To maintain a vanishing expansion at 0 K as well as a horizontal tangent at 0 K, in accordance with the approach of Röttger *et al.* to fitting the thermal expansion of water at low temperatures (Röttger *et al.*, 1994 ▸), the coefficients *A*_1_ and *A*_2_ were set at zero. After fitting this yielded the relation:
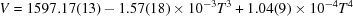
for methane II between 6 and 20 K. When fitting methane I between 22 and 80 K, the vanishing expansion and horizontal tangent at 0 K restraints are not relevant. Methane I was fit to a second-order polynomial [Fig. 2 ▸(*b*)], which resulted in the relation:
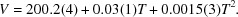
A challenge when fitting the volume data of methane I was the departure from the continuous trend between 65 and 75 K. This suggests that, at these temperatures, some larger crystalline grains are being formed.

**Figure 3:**
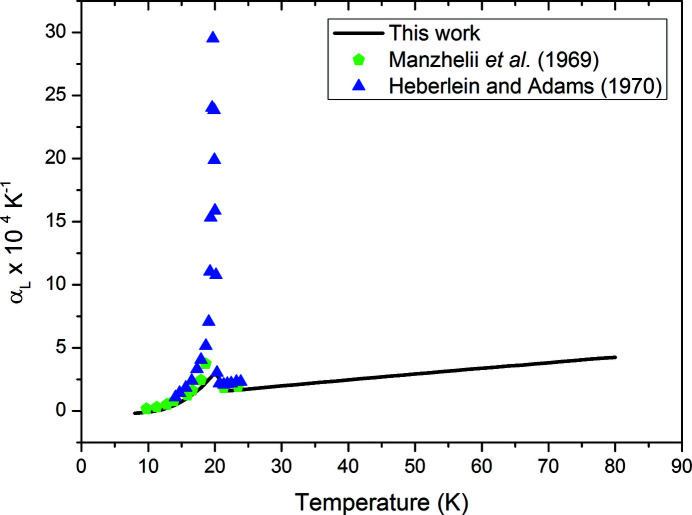
Comparison of the change in linear expansion coefficient with temperature (derived from neutron diffraction measurements of methane) with that from the work by Manzhelii *et al.* (1969 ▸) and Heberlein & Adams (1970 ▸).

We have taken the derivative of the volume expansion to determine the linear expansion coefficient and then compared our results with previous measurements in Fig. 3 ▸ using the following relation:
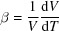
and
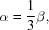
where β is the volume expansivity and α is the linear expansivity. The previous linear measurements for methane were undertaken with a dilatometer, and over a restricted temperature range (Heberlein & Adams, 1970 ▸; Manzhelii *et al.*, 1969 ▸). Our results are very much in agreement with the previous determinations, except for the very large spike in linear thermal expansion observed at the phase II to phase I transformation by Heberlein & Adams, reaching a maximum of 0.0296 K^−1^. This is likely to be due to the fact that our temperature steps were not as fine as in the previous investigation; we have not plotted all the points from the work by Heberlein & Adams, but they were generally in intervals of  0.15 K, compared with our temperature intervals of 2 K. It should also be noted that the points charted from previous work are numerical derivatives from their data, whereas this work uses an analytical derivative from the fitted volume expansion polynomial. Our results followed a similar trend to the work of Manzhelii *et al.*, which showed a similar maximum in linear thermal expansion (0.00374 K^−1^) as that determined by this experiment (0.00299 K^−1^) from data collected in 1 K intervals. Such a large change in expansion at the transition is not unexpected, and has interesting implications where the methane landscapes traverse these temperatures. However, as Pluto’s temperatures are expected to reach a minimum of 24.4 K (Earle *et al.*, 2017 ▸), further study of this expansion maximum is unlikely to be relevant to this dwarf Planet.

It should also be noted from Figs. 2 ▸(*a*) and 3 ▸ that, between 8 and 10 K, methane exhibited negative thermal expansion. Though we do not have finer temperature steps to confirm this, it does support the work of Heberlein & Adams (1970 ▸) who also noted this in the relative length changes of their methane sample.

### 3.2. Nitrogen   

#### 3.2.1. Thermal expansion of nitrogen between 6 and 64 K   

**Figure 4:**
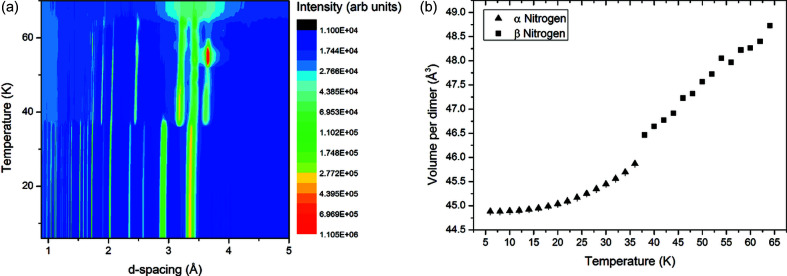
(*a*) Thermodiffractogram of neutron diffraction data collected on nitrogen between 6 and 64 K, intensity is plotted with a log_10_ scale to visualize weaker features. (*b*) Extracted volume per molecule from a Pawley fitting of the nitrogen diffraction. Error bars in the measurement are plotted but are generally smaller than the data points. The fitted lattice parameters used to generate (*b*) are tabulated in the supporting information.

Unlike methane, no incoherent hydrogen scattering is present in neutron diffraction from nitrogen. Thus, our data from nitrogen were of excellent quality and consequently allowed us a number of insights into the structure of nitrogen as well as determining the volume behaviour with temperature. The data collected are presented in Fig. 4 ▸ and can be downloaded via https://doi.org/10.5281/zenodo.3635106 and https://doi.org/10.5281/zenodo.3635103.

Like methane, solid nitrogen has two forms (elemental allotropes) at ambient pressure, labelled α and β. The high-temperature β structure forms a hexagonal close-packed structure with the *c*/*a* ratio close to the ideal value for close-packed spheres. This arises from the disorder of the N_2_ molecules and although some early X-ray diffraction measurements (Streib *et al.*, 1962 ▸) suggested that the N_2_ molecules would precess about the *c* axis, later work (Press & Hüller, 1978 ▸) showed that this was not the case, and that the nitrogen dimers would be completely spherically disordered. As discussed subsequently, there has also been some debate over the low-temperature α-nitrogen crystal structure, but the most cited model is that where the structure is cubic 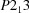 with four molecules in the unit cell.

**Figure 5:**
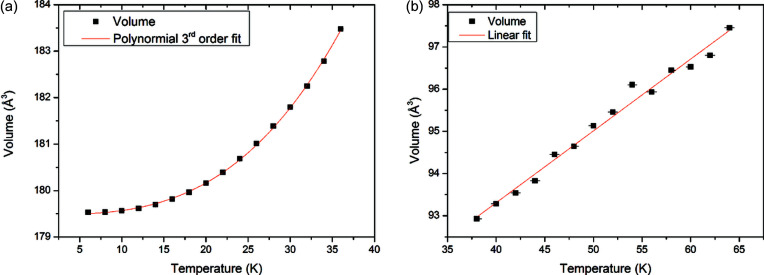
Polynomial fits to volume data from (*a*) α-nitrogen and (*b*) β-nitrogen. The expressions for the fits are described in the text and the fitted lattice parameters used to generate these figures are tabulated in the supporting information.

**Figure 6:**
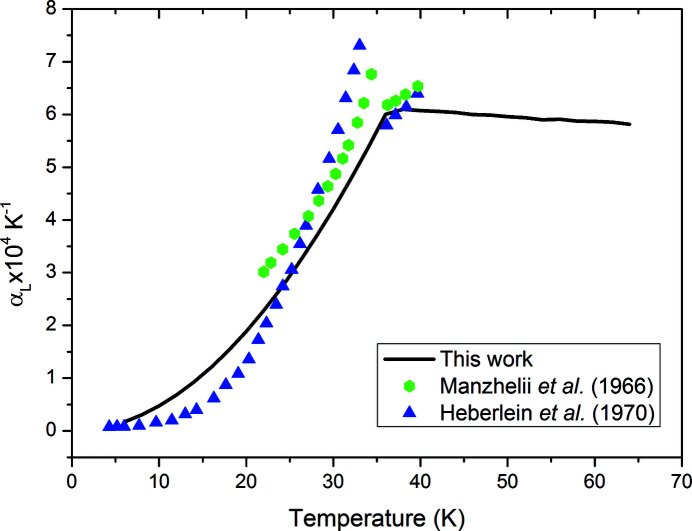
Comparison of the temperature dependence of the linear expansion coefficient from our neutron diffraction measurement with the work of Manzhelii *et al.* (1966 ▸) and Heberlein *et al.* (1970 ▸).

Fitted volume data presented in Fig. 4 ▸(*b*) are also tabulated in the supporting information. We have approached fitting these data in the same way as the methane data, not wishing to overparameterize. We have fitted the α-nitrogen data with the same constraints as those we applied to methane phase II, that is, to maintain a vanishing expansion at 0 K as well as a horizontal tangent at 0 K, the coefficients *A*_1_ and *A*_2_ of the polynomial fit were set at zero. As we saw no improvement in reduced χ^2^ between a third- and fourth-degree polynomial fit, the final fit is
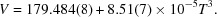
For β-nitrogen, like methane phase I, we applied no constraints to the polynomial terms. As the reduced χ^2^ did not improve when adding higher-order terms, a linear fit was sufficient:
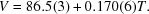
These fits are presented with the data in Fig. 5 ▸. Like methane, the linear thermal expansion of nitrogen has previously been determined with a dilatometer over a limited range: 4–40 K in the work by Heberlein *et al.* (1970 ▸) and 20–44 K in the work by Manzhelii *et al.* (1966 ▸). Fig. 6 ▸ compares our measurements with these previous works using the same method of derivative calculation as the methane study.

Our measurements again do not show as large a maximum in the linear expansion compared with that of previous work, although the difference is less stark. Broadly, our measurements compare rather well with the work of Manzhelii *et al.* and Heberlein *et al.* below the transition to β-nitrogen at 35.6 K. At temperatures above this point, both previous measurements suggest that the linear expansion coefficient of nitrogen should increase with temperature. In this work, as the temperature dependence of the volume of β-nitrogen has been fitted with a linear trend, there is a slight decrease in linear expansivity (α_L_).

#### 3.2.2. Refinement of the α-phase of nitrogen   

**Figure 7:**
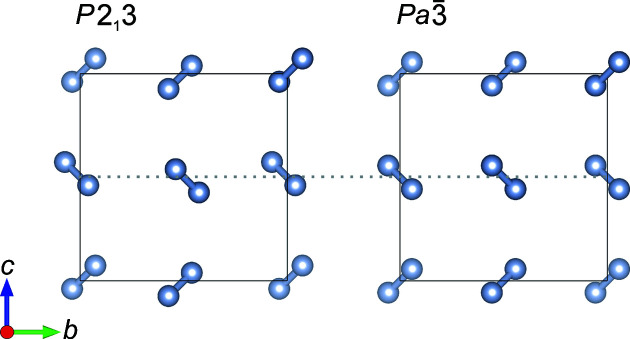
Comparison of the two models of the α-nitrogen structure, one with the centre of the nitrogen molecules offset from the origin (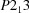) and one with the centre of the molecules at the origin (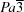). The grey dotted line is included as a guide for the eye.

Given the quality of our data and that no neutron diffraction measurements of α- or β-nitrogen have been published, we pursued the analysis of the crystal structures of nitrogen. (Kjems & Dolling (1975 ▸) grew a single crystal of N_2_ for an inelastic neutron study and chose to interpret their results on the basis of the the 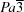 structure.) The structure of the ordered α-phase has been subject to intense debate since the 1960s, with the difference between the two models illustrated in Fig. 7 ▸.

This has principally centred around the assignment of the space group to symmetric 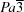, where the centre of the nitrogen molecules lies at the origin; or to non-centrosymmetric 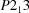, in which the centre of the nitrogen molecules is offset by ∼0.16 Å  from the origin. 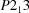 has become the more accepted crystallographic structure, based on data from a solitary single-crystal X-ray diffraction study by Jordan *et al.* (1964 ▸); a later study used the measured intensities from this study to further refine the α-structure (La Placa & Hamilton, 1972 ▸). Jordan *et al.* justified this assignment with weak observations of [520] and [501] reflections, which are not allowed in the 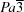 space group. The 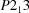 structure has been supported by zero-field NMR studies (Brookeman *et al.*, 1971 ▸), in which the authors also report the presence of piezoelectricity (Brookeman & Scott, 1972 ▸), a property inferred from the non-centrosymmetry of the 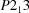 structure. On the other hand, the 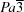 structure is supported by Raman spectroscopy [for instance, Anderson *et al.* (1970 ▸)] and electron diffraction measurements (Venables & English, 1974 ▸), and has been pursued by the theoretical community due to doubts over the 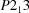 structure (Kjems & Dolling, 1975 ▸).

Initial structural analysis of the data from which Fig. 4 ▸ was derived indicated that the accepted 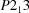 structural model is not preferred over the 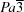 structure. Hence, we undertook a separate data collection from a fresh sample at 2.413 (1) Å (https://doi.org/10.5281/zenodo.3635103). After verifying that the sample was a well averaged powder using the two-dimensional images from the detector, we refined five models against the data. As well as the model parameters displayed in Table 1 ▸, lattice parameters and crystalline size were also allowed to vary, as well as a four-term spherical harmonic model for preferred orientation included at the last stage. The five models are as follows:

(*a*) Model 1, space group 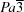, has one nitrogen atom position (N1) with the position refined along with an isotropic displacement parameter.

(*b*) Model 2, space group 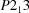, has two nitrogen atom positions (N1 and N2) fixed to the positions determined by Jordan *et al.* (1964 ▸), refined with isotropic displacement parameters.

(*c*) Model 3 is identical to model 2, but with the nitrogen positions allowed to refine.

(*d*) Model 4, space group 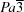, has one nitrogen atom position (N1) with the position refined along with anisotropic displacement parameters.

(*e*) Model 5, space group 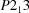, has two nitrogen atom positions (N1 and N2) refined along with anisotropic displacement parameters.

Table 1 ▸ provides the results of fitting these five models against the data collected at 16 K. In Model 3, where the 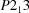 nitrogen atom positions are allowed to vary, they move to positions very close to the atomic position in the 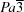 model (Model 1) and exhibit a correlation coefficient of 0.89. On this basis, rejection of the 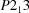 structure is advisable. As might be expected, the models with anisotropic displacement parameters resulted in slightly better fits for the 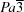 and 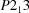 models to the data, but the difference in *wR*_p_ between the 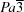 and 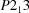 structures, given the extra parameters, is not significant.

**Figure 8:**
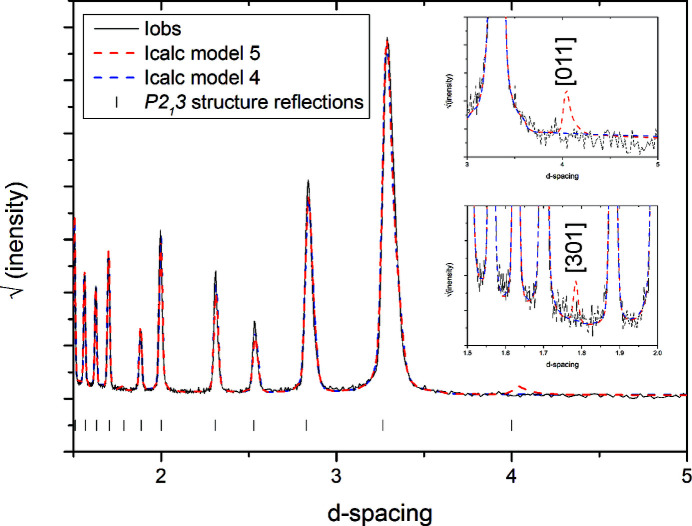
Rietveld fits of Model 4 and Model 5 to α-nitrogen data collected at 16 K and 2.413 (1) Å. Details of the fits are given in Table 1 ▸. Inset are details of the diffraction pattern and models, showing the absence of the [011] and [301] reflections.

Hence, as none of the models based on 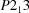 show a significantly better fit compared with the 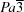 models, the simpler model must be the preferred description of the α-nitrogen structure. The data fits in Fig. 8 ▸ support this preference, as the 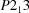 structures allow for there to be intensity in both the [011] and [301] reflections. These reflections are absent in the measured data and systematically absent in the space group 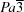. Hence, we conclude that the 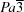 structure is the more accurate description of the α-nitrogen structure, rather than the currently widely cited 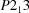 structure. We note that the previous single-crystal investigation (Jordan *et al.*, 1964 ▸) did not consider the possibility that multiple diffraction could have caused the weak observations of the [520] and [501] reflections, which were the only violations of the 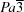 symmetry they noted.

### 3.3. Refinement of the β-phase of nitrogen   

**Figure 9:**
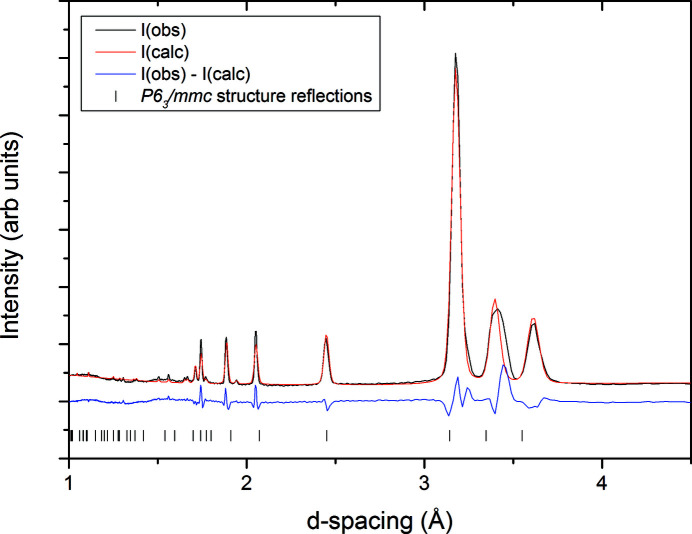
Rietveld refinement of the β-nitrogen structure to the data collected at 1.641 (1) Å and 38 K, *wR*_p_ 8.4%. The poor fit of the [002] peak suggests that, even shortly after the transformation from the α-form, the sample of β-nitrogen shows some strain.

Rietveld refinement was also performed on the nitrogen data collected at 1.641 (1) Å at 38 K, when the sample had fully transformed to the β-form. We proceeded to use the model of the β-nitrogen structure that describes the disorder of the nitrogen molecule with the atom distributed about a 24-fold site with 1/12th occupancy (Streib *et al.*, 1962 ▸). As well as refining the nitrogen atom position, we also allowed an isotropic atomic displacement parameter and the lattice parameters to refine. The resultant fit to the data is presented in Fig. 9 ▸.

The fit results in a nitrogen atom position at fractional co-ordinates *y* = 0.566 (13) and *z* = 0.289 (7), as well as an isotropic atomic displacement parameter of 0.19 (2) Å^2^.

### 3.4. Textural changes in the disordered phases of methane and nitrogen   

**Figure 10:**
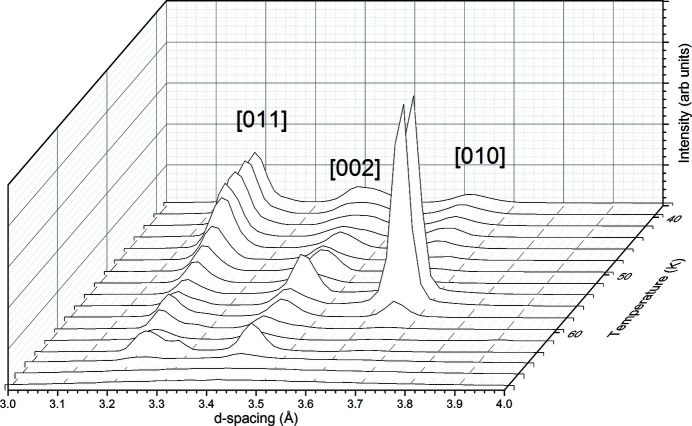
Details of the changes in relative intensity of the [011], [002] and [010] peaks of β-nitrogen on warming.

Both phase I of methane and β-nitrogen are described as ‘plastic’ crystals, where the weakly interacting nature of the molecules and the orientational freedom are thought to impart mechanical softness to the overall material. However, at present many of the mechanical properties of both methane and nitrogen remain elusive, although some insight may be gained from examining the data presented in this paper. Both methane and nitrogen exhibited changes in relative intensities of the peaks on warming which we infer are due to increasing grain size and the average of the bulk orientation changing as a result of this. It is harder to discern in the methane data [Fig. 1 ▸(*a*)] but can be clearly seen in the nitrogen data presented in Fig. 4 ▸(*a*). Fig. 10 ▸ shows a closer view of the nitrogen data, focusing specifically on the [011], [002] and [010] reflections.

On transformation from the α-form to the β-form above 36 K, we observed that the sample is a well averaged powder, based on both the two-dimensional images from the WOMBAT detector and the fact that Rietveld fitting to the data required no correction for preferred orientation. Fig. 10 ▸ shows that this is maintained until 48 K, where the relative intensities start to change. As warming continues from this point, the overall intensity of the peaks drops; but by 54 K there is a dramatic increase in the intensity of the [010] peak, indicating significant crystallite orientation along the *b* axis. This lasts only until the sample is warmed to 58 K, where the preferred orientation changes again. In fact, if the relative intensity changes from 48 K can be attributed solely to changing crystallite orientation, then there are significant changes with each temperature step. At present we infer that this effect occurs due to grain growth, and it can be seen to also affect the overall lattice parameters of the sample (Fig. 5 ▸). The effect of this, which we also observed in subsequent runs, could be informative for Plutonian glaciology; we are pursuing future studies to address the textural changes of nitrogen and methane with temperature.

## 4. Summary   

**Figure 11:**
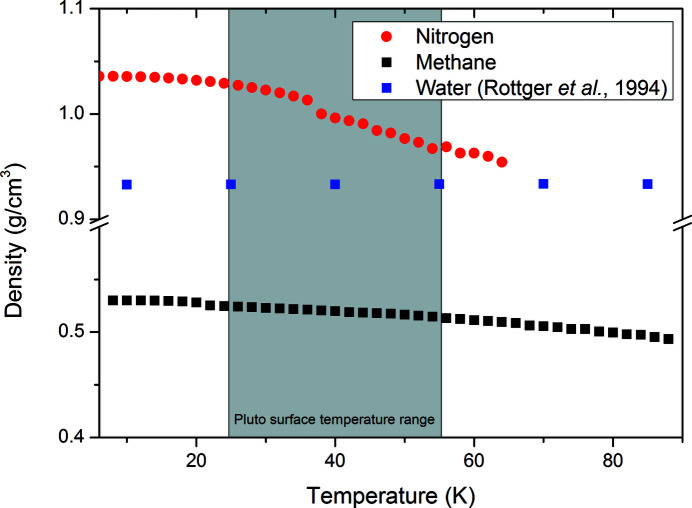
Comparison of the density changes with temperature of solid nitrogen, methane and water, with reference to the temperature extremes on the dwarf planet Pluto.

In light of the discoveries made by the *New Horizons* mission, we have conducted a re-examination of the solid crystalline behaviour of methane and nitrogen at low temperatures. Neutron powder diffraction has enabled us to chart the expansion behaviours of these materials across their range of solid phases for the first time. The benefits of undertaking this investigation with neutron diffraction are that we have been able to assign the structure of α-nitrogen to a 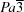 structural model and also note the textural behaviour of β-nitrogen and methane phase I on warming. Our results also enable us to calculate the density changes of both nitrogen and methane over the range of Pluto’s seasonal temperature variation. In Fig. 11 ▸, we present this along with the density of water over the same range.

As has been noted before, the large contrast in density between nitrogen and methane has implications for the possible layering of these materials on Pluto (Grundy *et al.*, 2016 ▸). What is quite interesting, though not unexpected in a hydrogen-bonded structure, is how little water expands over this temperature range. Furthermore, the α-nitrogen to β-nitrogen transition will potentially occur over large areas of nitrogen on Pluto’s surface. Both the density change and the grain size change are likely to have significant impact on Pluto’s glaciers and may explain some of their observed features.

## Supplementary Material

Crystal structure: contains datablock(s) I. DOI: 10.1107/S2052252520007460/zx5020sup1.cif

Rietveld powder data: contains datablock(s) global. DOI: 10.1107/S2052252520007460/zx5020sup2.rtv

Neutron powder diffraction from NIST 660c LaB6 standard for calibration of Wombat high-intensity neutron powder diffractometer: https://doi.org/10.5281/zenodo.3637634

Neutron powder diffraction data of solid methane in the range 8K-90K: https://doi.org/10.5281/zenodo.3634152

Neutron powder diffraction data from nitrogen in the range 16K-72K: https://doi.org/10.5281/zenodo.3635106

Neutron powder diffraction data from solid nitrogen in the range 6K-70K: https://doi.org/10.5281/zenodo.3635103

CCDC reference: 2007945

## Figures and Tables

**Table 1:** Final parameters of the five models of α-nitrogen refined against data collected at 16 K and 2.413 (1) Å For the anisotropic displacement parameters, only *U*_11_ and *U*_12_ were refined because of symmetry restraints on these sites.

Model	Space group	Site	*x* (Å)	*U*_iso_ (Å^2^)	*U*_11_	*U*_12_	*wR*_p_ (%)
1	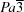	N1	0.0575 (2)	0.051 (1)	–	–	11.9
2	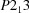	N1	0.0735	0.023 (2)	–	–	24.2
		N2	−0.0388	0.060 (2)	–	–	
3	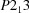	N1	0.047 (1)	0.044 (5)	–	–	11.7
		N2	−0.068 (2)	0.05 (1)	–	–	
4	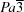	N1	0.057 (2)	–	0.051 (1)	0.003 (1)	11.8
5	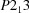	N1	0.074 (2)	–	0.06 (1)	−0.010 (4)	11.6
		N2	−0.042 (1)	–	0.043 (4)	−0.013 (6)	
